# Generating a Synthetic Lumbar CT from a Standard MRI Protocol

**DOI:** 10.3390/jcm14082809

**Published:** 2025-04-18

**Authors:** Yehiel Shilo, Frank M. Phillips, Timothy F. Witham, Kornelis Poelstra, Lital Hodeda, Moshe Stavsky

**Affiliations:** 1 MRI2CT, Jerusalem 9463121, Israel; 2 Department of Orthopedic Surgery, Rush University Medical Center, Chicago, IL 60612, USA; 3 Department of Neurosurgery, Johns Hopkins University, Baltimore, MD 21218, USA; 4 The Robotic Spine Institute of Las Vegas, Nevada Spine Clinic, Las Vegas, NV 89128, USA; 5 Department of Orthopedic Surgery, Shaare Zedek Medical Center, Facularty of Medicine, Hebrew University, Jerusalem 91120, Israel

**Keywords:** MRI to CT, synthetic CT, spine MRI, lumbar MRI, lumbar spine surgery, surgery planning, deep learning, artificial intelligence

## Abstract

**Background:** Eliminating the need for a preoperative spine CT scan prior to spine surgery offers significant financial advantages, reduced radiation exposure, and a more streamlined workflow. This can be achieved by converting a standard-protocol MRI scan into a synthetic CT (sCT), which provides a precise representation of bone structures. The sCT is intended for use in the preoperative planning and navigation of spine surgeries, eliminating the need for an additional CT scan. **Methods:** The transformation is based on a neural network architecture that converts MRI data into high-resolution sCT images. Although the resolution of standard magnetic resonance imaging ranges from 3 to 5 mm, sCT achieves sub-millimeter accuracy (below 1 mm). In this study, we present the results of the generation of sCT and compare them with conventional CT scans of the same patients. **Results:** A comprehensive comparison was conducted using both 3D and 2D measurements on 500 different vertebrae. The 3D evaluation used 3D surface distance measurement to assess the full vertebrae and key anatomical elements. The 2D analysis focused on critical distances that define the vertebral body, pedicles, and spinous processes. All measurements were performed automatically and validated by orthopedic surgeons, ensuring clinical relevance. **Conclusions:** This study shows that converting a standard-protocol MRI scan into an sCT provides a precise representation of bone structures.

## 1. Introduction

MRI is the diagnostic imaging modality of choice for evaluation of patients with complex spinal disorders [1], as supported by studies demonstrating its efficacy [2]. However, integrating MRI into surgical applications, particularly for fluoroscopy-guided procedures, presents challenges due to variability in spatial resolution and intensity distributions across manufacturers and clinical sites [2,3]. Conversely, CT remains the preferred imaging modality for planning and assessment in specific surgical contexts, particularly for image-guided surgeries [3,4]. Nonetheless, CT usage introduces significant patient radiation exposure, which clinicians strive to minimize [5,6], as well as being an additional logistical hurdle before surgery, increasing costs and wait times. Spinal fusion with pedicle screw instrumentation is widely performed to treat varied spinal pathologies. Historically, instrumentation is placed with fluoroscopic guidance to achieve accurate positioning of the instrumentation. The integration of medical imaging with advances in computational technology has facilitated stereotactic neuronavigation and its incorporation with robotic systems, revolutionizing spine surgery. Image-guided spine surgery represents a critical breakthrough, enhancing both patient safety and surgical efficiency.

Intraoperative navigation is widely used during spine surgery to ensure precise placement of instrumentation [7] and requires use of a pre- or intraoperative CT. Accurate preoperative planning and detailed delineation of vertebrae from diagnostic imaging are fundamental tasks in image-guided procedures. Detailed characterization of vertebral morphology during the planning phase significantly aids in optimizing pedicle screw trajectories, avoiding critical anatomical structures, such as nerves and arteries, and minimizing potential complications [2].

In patients with spinal disorders, X-rays are often the primary diagnostic tool, with magnetic resonance imaging added as part of routine evaluation [8]. CT scans may be added to assess complex bony anatomy as well as in preparation for image-guided surgeries, which are increasingly being utilized worldwide. To address the limitations of current imaging modalities, this study explores the development of an artificial intelligence-based platform that generates a three-dimensional CT-like representation of the spine from MRI scans. The platform takes a standard MRI protocol as input and produces a high-resolution synthetic CT (artificial CT) as output.

The successful creation of an accurate CT-like structure from MRI would reduce reliance on CT scans, thereby decreasing patient exposure to ionizing radiation, alleviating the economic burden, and easing the strain on patients and healthcare systems. This innovation has the potential to transform the planning and execution of spine surgery while prioritizing patient safety and healthcare efficiency.

The generation of sCT from MRI has been explored in both radiotherapy applications [9] and orthopedic imaging. This is typically achieved using neural networks with various architectures, including convolutional neural networks (CNNs) [10] and transformers [11].

Until now, generating an sCT from an MRI scan required a specific MRI protocol that involved high-resolution imaging [10], which is both expensive and time-consuming. The current study evaluated the ability to create a synthetic CT with a standard MRI study to achieve levels that are sufficient for planning and navigation of spine surgery, eliminating the need for a separate, high-resolution MRI scan or any specialized sequence.

### MRI Protocols

MRI scans are performed slice by slice, making the process both time-consuming and expensive. To minimize scanning time, MRI is often conducted at a low resolution.

**Figure 1:**
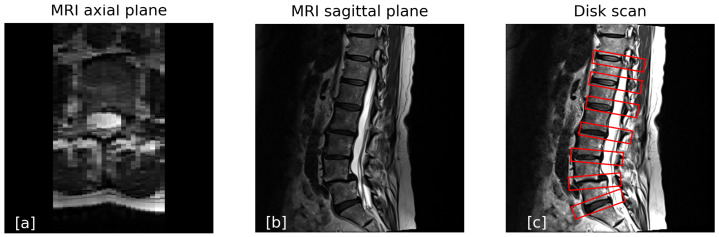
(**a**) The axial view of a T2 standard MRI. (**b**) The sagittal view of a standard MRI. (**c**) Sagittal view of the protocol for a skipping axial scan, in which only the intervertebral disks are scanned. The red boxes represent the parts that are scanned.

When a patient undergoes a standard MRI protocol, the process typically includes the following:

- A sagittal scan using both T1- and T2-weighted protocols. These scans have a low resolution in the left–right (LR) direction, with slice thicknesses ranging from 3 to 5 mm, depending on the site-specific protocol. In these cases the axial slice is not usable as its resolution is low, and only the sagittal slice is used, as seen in Figure 1.
- An axial scan, where the low resolution is along the inferior–superior (IS) axis. This scan is performed using one of two methods:
  1. Using a slice thickness of 4–6 mm, and therefore it is not usable for finding the exact bone structure.
  2. Scanning only the intervertebral disks while skipping the vertebrae, as shown in Figure 1c.

While the sagittal view in MRI provides good image quality, interpreting the axial view can be challenging due to its lower resolution.

The resolution limit is one of the main reasons why producing an sCT from MRI is a challenging task, and also why navigation cannot rely on MRI images. Previous works [10] have required a specific MRI protocol, with a 1 mm resolution. In the following work, we show an sCT that can be used for clinical usage and is based only on a standard MRI scan.

## 2. Materials and Methods

### 2.1. Generating Synthetic CT

The process of generating sCT is based on using neural networks that transform the MRI scan into a synthetic CT scan. A convolutional neural network (CNN)-based architecture was employed to generate the sCT. The model was trained on a large dataset comprising more than 1000 paired MRI and CT scans, all collected from patients with spinal conditions. The test cases were randomly selected and are representative of the overall population. The distribution of these test cases is shown in Table 1.

This is achieved by having a training process in which a low-resolution MRI is used as the input and a high-resolution scan of the CT is the output. The borders of the cortex are an important element for clinical planning purposes, and therefore this was the focus of our measurements. Hounsfield unit (HU) values were not included due to their clinical insignificance to this specific purpose. The time for converting a standard MRI scan into an sCT is less than 30 s.

### 2.2. Three-Dimensional Segmentation and Labeling

**Figure 2:**
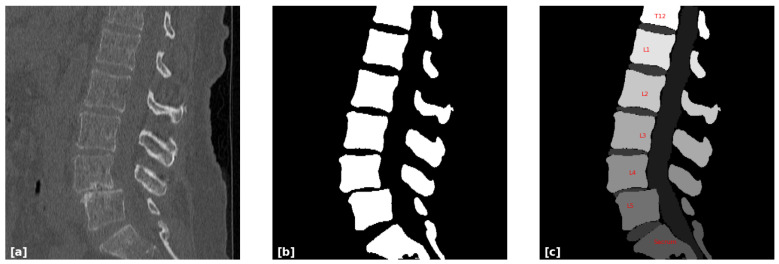
Segmentation and labeling process of a CT scan. (**a**) A sagittal slice from the original CT scan. (**b**) The 3D spine segmentation of the original CT scan on the same slice, in which all bone structures are white. (**c**) Labeling the different vertebrae, starting with the sacrum.

Before performing any measurements, a 3D spine segmentation is required to convert the scan (CT or sCT) into a binary format, where each voxel is classified as either bone or non-bone, as seen in Figure 2. This 3D segmentation is performed automatically with exceptionally high accuracy. The evaluation results demonstrate extremely high agreement between the segmentation output and ground truth (GT). The ground truth is based on manual evaluation of the segmented CT. For CT scans, this segmentation step is applied to the original data. In contrast, for sCT, no additional processing is necessary, as the module that creates the sCT inherently creates the segmentation in its output as well. The subsequent step involves labeling each vertebra to establish accurate correspondence between the CT and the sCT. This process was performed manually, with the sacrum serving as a clear reference point. The first vertebra above the sacrum, typically L5, was manually annotated to initiate the naming sequence. Once the initial vertebra has been identified, the name of the remaining vertebrae follows a straightforward and systematic approach.

**Figure 3:**
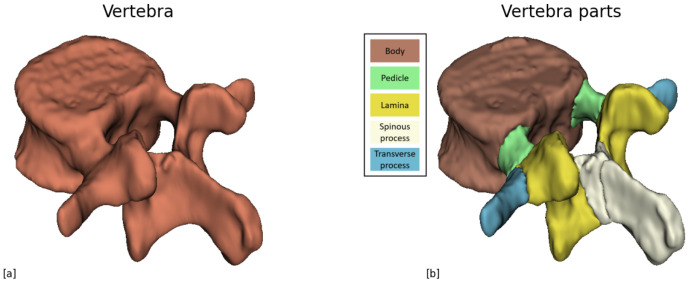
(**a**) A single vertebra after the 3D segmentation. (**b**) The same vertebra after the part segmentation has been applied. Each part is colored differently based on the legend.

The next step involves segmenting the vertebra into its anatomical components, including the vertebral body, pedicles, lamina, spinous process, and right and left transverse processes as illustrated in Figure 3. Since distinct boundaries between these elements are often lacking, achieving precise segmentation is challenging. Therefore, our measurements rely on segmentation as a reference to identify and extract the necessary data, rather than requiring complete accuracy.

The measurements are divided into two primary approaches: a detailed 3D comparison using the surface distance metric and a set of targeted length measurements. The ground truth is obtained from measurements performed on the original CT, and the comparison is made by applying the same measurements to the sCT.

### 2.3. Three-Dimensional Surface Measurements

For the 3D comparison, the surface of the vertebra needs to be used. The segmented 3D volume is converted into a mesh representation (a standard way to represent a 3D surface), which is then compared to the corresponding CT mesh. For each point on the sCT mesh, the minimum distance to the CT mesh is calculated, and then all points are averaged using the RMSE (root mean square error) method for representing the average distance between the two surfaces. Notably, this measurement is not symmetrical. This means that the surface distance result of comparing object A to object B (RMSE(sCT,CT)) will not be the same as the surface distance result of comparing object B to object A (RMSE(CT,sCT)). Therefore, RMSE(sCT,CT)≠RMSE(CT,sCT). In this specific study, the sCT is used as the reference point, and distances from the CT scan to the sCT are calculated. This means that every point in the CT is compared to the sCT which was derived from the patient’s MRI.

All measurements are conducted on individual vertebrae. Although each vertebra is a rigid structure with a fixed shape across different imaging modalities, such as CT and MRI, the relative positions and orientations of vertebrae can vary between scans. As a result, measurements involving multiple vertebrae are not presented as they differ between the scans.

**Figure 4:**
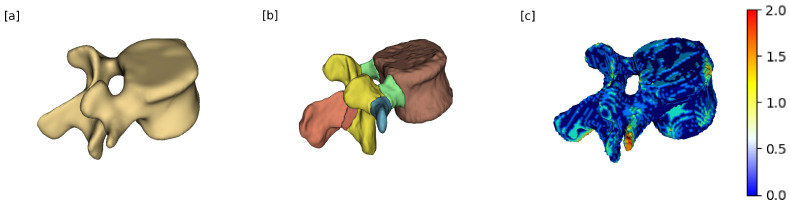
Illustration of how the surface distance measurements were performed. (**a**) The sCT vertebra. (**b**) The part segmentation of the same vertebra as the original CT. (**c**) The surface distance measurement of the entire vertebra, the color bar indicates the error associated with each point.

The surface distance analysis was performed on all lumbar vertebra for the full vertebra shape, and for specific anatomical components, including the vertebral body, the right and left pedicles, and the spinous process, as shown in Figure 4. These component-specific measurements used CT segmentation to identify anatomical elements, but distances were computed using all of the sCT.

### 2.4. Two-Dimensional Length Measurements

While 3D measurements can only be performed by a computer, we incorporate a set of 2D length measurements that can be manually verified by a human. Accurate vertebral measurements require a standardized alignment method for all vertebrae. To achieve this, a three-dimensional coordinate system is established using the following procedure: The upper end-plate plane is first identified to define the superior–inferior (SI) vector. The right–left (RL) vector is then determined by connecting the midpoints of the left and right pedicles. Once these two vectors are established, the anterior–posterior (AP) vector is automatically derived, completing the coordinate system. This standardized framework forms the basis for all subsequent measurements.

The measurement process begins by defining the appropriate measurement plane for each parameter. These planes are typically oriented obliquely to the original scan axes and are positioned according to specific anatomical requirements. Length measurements are then extracted from the segmentation data within these planes.

**Body measurements:** Vertebral body measurements are performed using the 3D part segmentation to isolate the vertebral body for accurate analysis. The following key measurement planes are employed:

- **Sagittal plane:** This plane divides the vertebral body into symmetrical halves, with its axes aligned along the AP and IS vectors. Within this plane, vertebral height is measured at both the anterior and posterior positions, which may not necessarily be parallel.
- **Axial planes:** Two axial planes are defined—one at the inferior end plate and another at the superior end plate of the vertebra—using the AP and RL axes. These planes facilitate the measurement of vertebral width and length, providing a comprehensive assessment of dimensional attributes.

**Figure 5:**
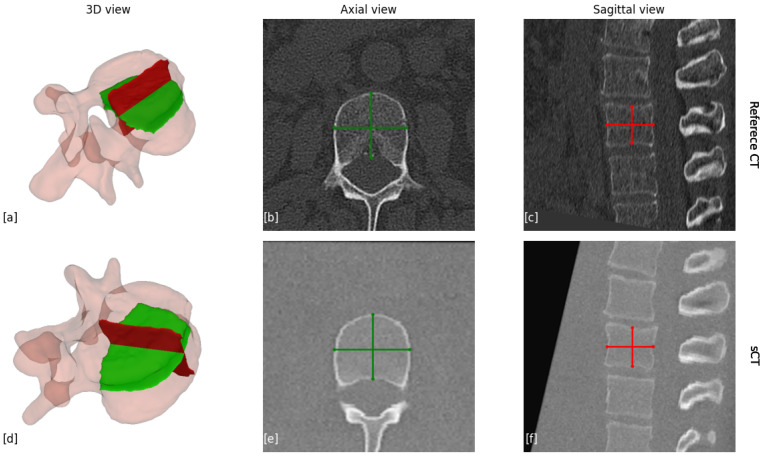
Measurements of the vertebra body. (**a**,**d**) Illustration of the 3D slices that were captured for measuring the body lines. (**b**) The axial view of the reference CT scan; (**c**) the sagittal view of the reference CT scan; (**e**) the axial view of the sCT scan; (**f**) the sagittal view of the sCT scan. The red lines present the line that was measured.

These measurements are illustrated in Figure 5.

**Pedicle measurements:** Pedicle measurements focus on accurately determining the width and height of each pedicle, following the same protocol for both the left and right sides. Using 3D part segmentation, individual pedicles are isolated, and the optimal plane for potential screw placement is identified. The measurement protocol involves three key steps:

1. Identification of the optimal pedicle screw trajectory;
2. Selection of the narrowest cross-sectional plane along this trajectory;
3. Measurement of pedicle width and height within this plane.

**Figure 6:**
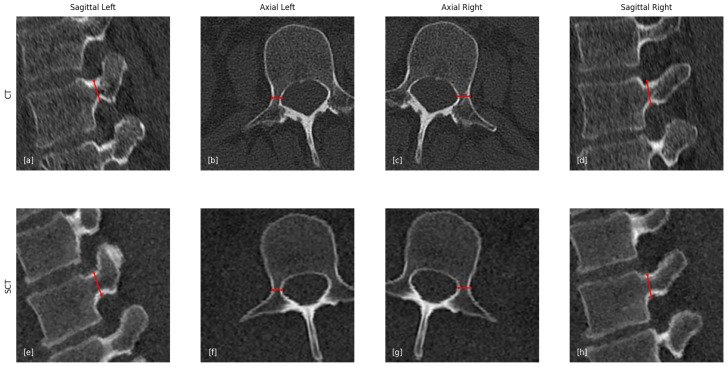
Comparison of the pedicle measurement between CT and sCT. The red line indicate the measurement that was performed. (**a**,**e**) CT/sCT sagittal view of left pedicle. (**b**,**f**) CT/sCT axial view of left pedicle. (**c**,**g**) CT/sCT axial view of right pedicle. (**d**,**h**) CT/sCT sagittal view of right pedicle. The red lines present the line that was measured.

This standardized method ensures consistent, clinically relevant measurements for surgical planning and analysis. The width and height are measured for both pedicles, as illustrated in Figure 6.

**Figure 7:**
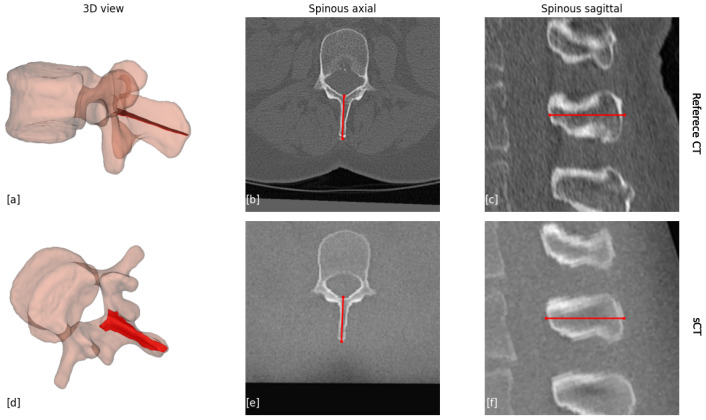
Measurements of the spinous process line. (**a**,**d**) Illustration of the 3D slice that was captured for the spinous process measurement. (**b**) The near-axial view of the reference CT scan; (**c**) the sagittal view of the reference CT scan; (**e**) the near-axial view of the sCT scan; (**f**) the sagittal view of the sCT scan. The red lines present the line that was measured.

**Spinous process measurements:** For the spinous process, the measurement involves identifying the longest straight line in the spinous process, as seen in Figure 7. This is achieved by finding the oblique plane for which its center line in the AP direction is the longest.

## 3. Results

### 3.1. Baseline Characteristics of Patients

The above analysis was performed for 121 different cases, which included more than 500 different vertebrae. As explained in Section 2, all measurements were performed independently for both the original CT and the sCT. The analysis was performed only for the lumbar vertebrae. Cases with vertebral fractures or screws within the vertebra were excluded from the study.

In Table 1, patients are divided into sex and age distributions. The regional distribution of the patients can be seen in the lower part of Table 1. The literature indicates well-established anatomical differences between males and females, such as a greater tendency for a more lordotic lumbar spine in females, likely due to pregnancy and childbirth [12]. We analyzed the data and compared the accuracy of sCT to CT for the entire vertebral body RMSE measurement in relation to gender, finding no significant difference (*p* value = 0.18).

### 3.2. Visual Results of sCT from MRI

**Figure 8:**
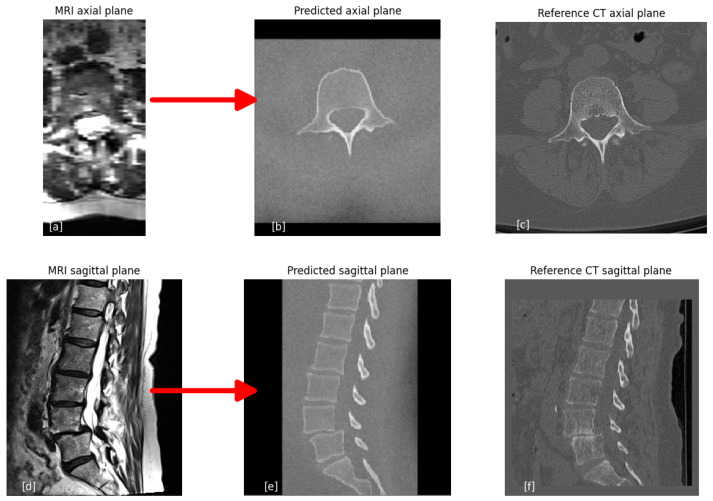
Conversion from MRI to sCT. Example slice from the input MRI T2 protocol of the (**a**) axial and (**d**) sagittal slices. The output sCT based on the input MRI is presented, viewing the (**b**) axial and (**e**) sagittal slices. Right: The reference CT of the same patient with similar (**c**) axial and (**f**) sagittal slices.

Figure 8 illustrates an example of the conversion process, showcasing axial and sagittal slices from the input MRI. The synthetic CT (sCT) is generated exclusively from the MRI scan, with the reference CT included solely for comparison purposes. As depicted, the sCT reproduces only the vertebral bones. The sCT consistently provides a clear visualization of the bone structures in all scenarios.

### 3.3. Assessment of Accuracy: Original CT Compared to sCT Parameters

**Figure 9:**
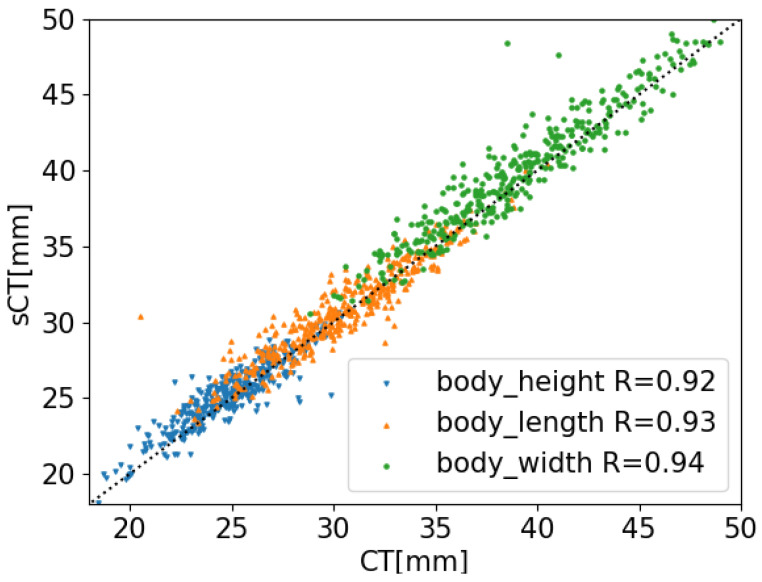
Scatter plot shows the correlation between sCT measurements and original CT measurements of the vertebral body. Pearson’s correlation is shown in the bottom of the plot; *p* value < 0.0020. The dotted line is a guide to the eye.

The analysis was conducted on patients with both CT and MRI scans, using CT-derived measurements as the baseline for comparison. The accuracy of the model was evaluated using both 3D and 2D measurements as shown in Table 2 and in Figure 9. The correlation between the sCT measurements and original CT measurements of the vertebral body was assessed using Pearson’s correlation coefficient with a *p*-value < 0.001. The 3D measurements, detailed in Section 2.3, were based on surface measurements and revealed a difference of approximately 0.5 mm between the sCT and CT results. The 2D measurements, described in Section 2.4, showed the same accuracy, with the exception of the body width and spinous process measurements, possibly attributable to their unique shapes. Overall, the results indicate that the vertebral structure is well preserved, with the lengths of various measurable components remaining consistent.

### 3.4. Assessment of Accuracy: The Presence of Radiologic Anatomic and Pathologic Findings

All data were collected from patients with various medical conditions. A thorough review was conducted to classify each scan based on specific medical conditions. These conditions are categorized into two groups: general spine-related issues and findings specific to individual vertebrae. Vertebra-specific issues are listed in the upper part of Table 3, while general spine-related conditions are shown in the lower part. This analysis highlights the potential applicability of sCT for these medical conditions.

**Figure 10:**
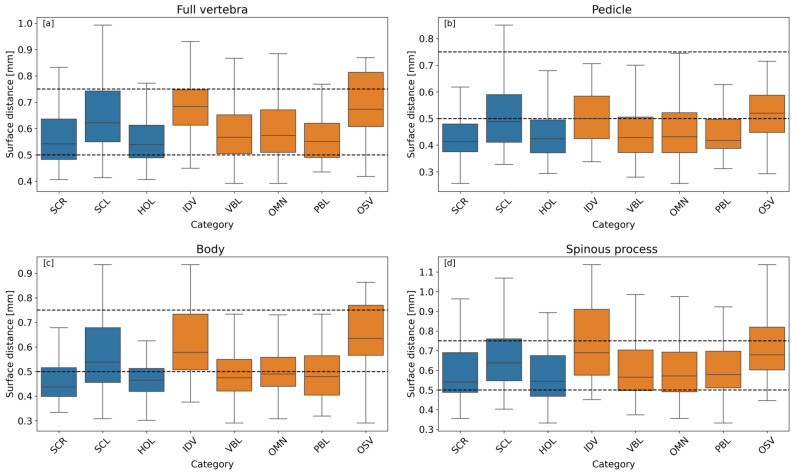
Boxplot of the surface distance as a function of the main clinical attributes. The impact of the different categories on the (**a**) full vertebra, (**b**) pedicle, (**c**) vertebra body, and (**d**) spinous process. The boxes in blue indicate a spine-related issue, and the boxes in orange indicate a vertebra-related issue. The abbreviations can be found in Table 3. Dotted lines are just a guide for the eye.

During the analysis, the impact of different anatomical conditions was evaluated. These conditions were grouped into two main categories: spine-related issues and vertebra-specific issues. Their distribution is presented in Table 3, while Figure 10 illustrates the effect of each condition. Anatomical conditions with fewer than 20 occurrences (2%) are excluded from the figure. The impact on surface distance values was assessed for the entire vertebra, vertebral body, pedicle, and spinous process. Among spine-related conditions, patients with scoliosis exhibited slightly lower performance, with surface distance values approximately 0.1 mm higher across all anatomical regions. Among vertebra-related conditions, patients with intervertebral disc vacuum (IDV) exhibited slightly lower performance, with surface distance values approximately 0.1 mm higher across all anatomical regions. In all specific anatomical conditions, the error never exceeded 1 mm.

**Figure 11:**
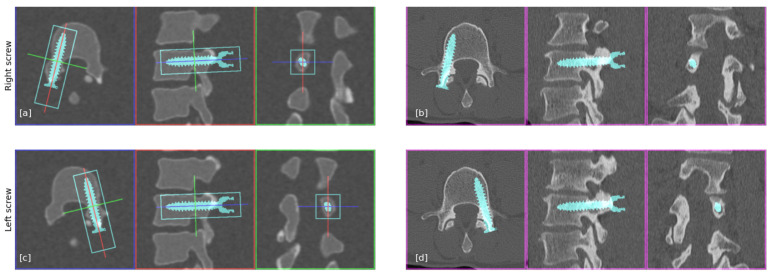
The planned screws on the sCT, and the way they will actually look in the real CT. Each sub-figure shows the screw placed in the 3 planes (axial, sagittal, and coronal). The colored lines are used to show the other planes according to the color. (**a**,**c**) Right/left screw as planned in the sCT. (**b**,**d**) Right/left screw as it will actually be placed in the CT.

As discussed above, the end point of this technology is preoperative planning and navigation in lumbar spine surgery. We made an example of surgical planning with pedicle screws on an sCT and than superimposed it on the specific patient’s real CT to emphasize the ability of accurate preoperative planing and navigation, as shown in Figure 11. The reason for the small variations between the sCT and the CT is the registration error between the two scans.

## 4. Discussion

The technology we have presented enables the use of synthetic CT (sCT) instead of traditional CT for surgical planning and navigation. Utilizing an MRI scan as the input for planning and navigation has the potential to significantly transform spine surgery workflows.

**Remarkable accuracy of sCT:** Studies [14,15] have shown that the maximum translational error tolerance for image-guided screw placement is 1 mm for each spine level. This means that with a surface distance error of less than 1 mm, screw placement would still be within a safe range. The maximum tolerated error should be less than 1 mm. In our model, a less than 1 mm accuracy was shown across a multitude of patient demographics, for different general spine-related pathologies, including scoliosis and disc vauum phenomena, and specific anatomical abnormalities categorized by individual anatomical vertebral components. This precision is particularly noteworthy given the complexity and variability of spinal anatomy, further solidifying sCT’s potential as a valuable tool for clinical assessment and surgical planning.

By adopting a standard MRI protocol instead of an additional CT scan, several advantages can be achieved.

**Radiation reduction:**

1. Eliminating preoperative CT radiation: Since CT scans involve relatively high radiation exposure, replacing them with sCT can significantly reduce radiation exposure. This is particularly crucial for young adults with scoliosis, who often undergo multiple imaging procedures, but is true for all patients.
2. Reducing intraoperative radiation exposure: In cases where a preoperative CT is not performed, an intraoperative CT is often used instead. By replacing this step with sCT, radiation exposure is minimized not only for the patient but also for the surgical team; another advantage in this case is the reduction in surgical room time and anesthesia time.

**Reduction in lead time:**

Eliminating the need for a CT scan streamlines the surgical preparation process.

1. For patients requiring a preoperative CT, significant time is saved by removing the steps of obtaining a referral, scheduling the scan, and waiting for results to be reviewed by the physician.
2. In cases where an intraoperative CT is used instead of a preoperative CT, avoiding this step reduces the time spent in the operating room (OR), ultimately improving OR efficiency and throughput. Additionally, intraoperative scans often have a limited field of view, sometimes requiring repeated scans to capture the necessary anatomical region. Avoiding this repetition further optimizes the surgical workflow.

Moreover, there is a significant financial advantage associated with eliminating the need for a CT scan, as well as considerable time-saving benefits. The time required to convert a standard MRI scan into an sCT is less than 30 s. This efficiency can translate to substantial cost savings for hospitals and patients, while also expediting surgical planning and navigation.

**Expanding the use of procedure planning and robotics-assisted spine surgery:**

For patients who previously could not benefit from preoperative planning and navigation-assisted surgery due to the absence of a CT scan, the use of sCT makes planning and robotic navigation more accessible. This advancement broadens the scope of robotics-assisted spine surgeries, enhancing precision and surgical outcomes.

**No need for specific MRI protocol:**

MRI is well known for its excellent soft tissue-imaging capabilities, while bone and mineralized tissues are more effectively visualized in CT scans. Artifacts resulting from patient movement during the MRI scan, as well as limitations in MRI technology [16], can influence image quality. These factors can, in turn, impact the accuracy of the sCT measurements. A previous study [17] has shown, in a cadaveric study, that sCT can be used based on a specific MRI protocol. Using our solution eliminates the need to perform a unique MRI scan with a specific protocol, with an excellent accuracy of less than 1 mm. We can use any type of MRI scanner and the conversion technology will create an sCT that can then be used in the preoperative planning process for spinal navigation that could be married up with any navigation system (robotics, standard nav, augmented reality, etc.).

**Limitations and disadvantages:**

This technology is not intended to replace CT scans for diagnostic purposes; its primary use is for surgical planning and navigation. Additionally, it currently relies solely on bony structures and not on nerves, blood vessels, or other anatomical structures. A future goal is to enhance this technology or develop new technology that can accurately segment soft tissues as well. Such an advancement would benefit various surgical fields, including free flaps, plastic surgery, and neurosurgery.

## 5. Conclusions

We have shown that converting a standard-protocol MRI scan into a synthetic CT (sCT) provides a precise representation of bone structures. We have demonstrated the potential of sCT to replace traditional CT scans, offering a reliable and efficient solution for the planning and navigation of spine surgery. By choosing this method, we can eliminate the need for preoperative CT scans, reducing radiation exposure and improving the streamlined workflow.

## Figures and Tables

**Table 1:** Patient age and regional distribution by sex. The numbers represent the total count, while the values in parentheses indicate the percentage of the total population. Due to rounding, the sum of all percentages may not appear to be exact.

Age and Region			
**Age [Years]**	**# All Patients (%)**	**# Male (%)**	**# Female (%)**
≤30	12 (10)	6 (5)	6 (5)
31–40	13 (11)	4 (3)	9 (7)
41–50	25 (21)	12 (10)	13 (11)
51–60	25 (21)	10 (8)	15 (12)
61–70	23 (19)	9 (7)	14 (12)
71–80	12 (10)	6 (5)	6 (5)
>80	11 (9)	4 (3)	7 (6)
Region	# All patients (%)	# Male (%)	# Female (%)
Asia	14 (12)	6 (5)	8 (7)
Europe	23 (19)	11 (9)	12 (10)
Middle East	59 (49)	25 (21)	34 (28)
US	25 (21)	9 (7)	16 (13)
Total	121	51 (42)	70 (58)

**Table 2:** The upper section of the table displays the surface distance measurements, while the lower section presents the 2D measurements. For each parameter, the median, standard deviation, and the 75th and 95th percentile values are provided.

CT vs. sCT Measurements				
**Surface Distance Measurements**	**Median [mm]**	**std [mm]**	**75% [mm]**	**95% [mm]**
Full vertebra	0.567	0.199	0.675	1.052
Pedicle	0.428	0.168	0.673	0.805
Body	0.494	0.171	0.575	0.869
Spinous process	0.558	0.197	0.0.675	0.968
2D measurements	median [mm]	std [mm]	75% [mm]	95% [mm]
Pedicle width	0.452	0.55	1.244	1.614
Pedicle height	0.415	0.509	1.132	1.482
Body length	0.518	0.486	0.975	1.613
Body height	0.691	0.561	1.058	1.876
Body width	0.922	0.743	1.571	2.478
Spinous process length	1.035	1.074	1.84	3.334

**Table 3:** Breakdown of anatomical issues. The data are categorized into two groups: vertebra-specific issues and general spine issues. The numbers represent the total number of cases, while the values in parentheses indicate the percentage of the respective category population.

**Vertebra Conditions**							
**Condition**	**Abbr.**	**L1**	**L2**	**L3**	**L4**	**L5**	**Sum**
Vertebrae Body Lesion	VBL	55 (7)	51 (6)	63 (8)	59 (8)	53 (7)	281 (36)
Osteophyte minor [13]	OMN	45 (6)	53 (7)	64 (8)	65 (8)	50 (6)	277 (35)
Osteophyte severe	OSV	12 (2)	15 (2)	12 (2)	11 (1)	14 (2)	64 (8)
Pedicle Bone Lesion	PBL	8 (1)	11 (1)	15 (2)	22 (3)	14 (2)	70 (9)
Intervertebral Disc Vacuum	IDV	13 (2)	13 (2)	9 (1)	20 (3)	14 (2)	69 (9)
Process Lesion	PRL	7 (1)	3 (0)	4 (1)	4 (1)	7 (1)	25 (3)
Spondylolisthesis	SPL	0	0	0	4 (1)	2 (0)	6 (1)
Total		140 (18)	146 (18)	171 (21)	183 (23)	158 (20)	786 (100)
Spine conditions							
Condition	Abbr.	# Patients					
Scoliosis	SCL	29 (42)					
Hypo-Lordosis	HOL	21 (31)					
Sacralization	SCR	15 (22)					
Lumbarization	LMB	3 (4)					
Hyper-Lordosis	HRL	1 (1)					
Total		69 (100)					

## Data Availability

The datasets (i.e., the MRI and CT scans) presented in this article are not readily available because the data have ongoing technical and time limitations. Requests to access the datasets should be directed to yehiel@mri2ct.ai.
